# Diploma Vending

**Published:** 1881-07

**Authors:** 


Diploma Vending.—In this history seems to have repeated itself, for the recent details of Buchanan's confession are fully paralleled in the Indiana Christian College of fifty years ago. This institution, like the parent Buchanan institution, was chartered for theological and philanthropical purposes. It had its agencies in other States, notably in New York City, where two regular physicians were disciplined by the County Medical Society for being concerned in the nefarious business. Buchanan, in the extent of his operations, went far beyond this prototype, for he sold twenty thousand diplomas in the United States and forty thousand in Europe.

The principal agent in the European diploma-vending was a Revered, one of the prominent "English Medical Reformers," and the kind of reform he wanted might be judged by his principal occupation. The whole business simply emphasises what was said in the last number of the *Review*, respecting medical education. Had there been a system of State examinations entirely independent of the colleges, the need of a diploma would not have been so urgent, and diploma-vending would not have been so profitable a trade.—*Chicago Medical Review.*

